# Lower Limb Locomotion Activity Recognition of Healthy Individuals Using Semi-Markov Model and Single Wearable Inertial Sensor

**DOI:** 10.3390/s19194242

**Published:** 2019-09-29

**Authors:** Haoyu Li, Stéphane Derrode, Wojciech Pieczynski

**Affiliations:** 1 LIRIS, CNRS UMR 5205, École Centrale de Lyon, 69130 Ecully, France; stephane.derrode@ec-lyon.fr; 2 SAMOVAR, CNRS UMR 5157, Telecom SudParis, Institut Polytechnique de Paris, 91011 Evry CEDEX, France; wojciech.pieczynski@telecom-sudparis.eu

**Keywords:** gait analysis, lower limb locomotion activity, triplet Markov model, semi-Markov model, on-line EM algorithm

## Abstract

Lower limb locomotion activity is of great interest in the field of human activity recognition. In this work, a triplet semi-Markov model-based method is proposed to recognize the locomotion activities of healthy individuals when lower limbs move periodically. In the proposed algorithm, the gait phases (or leg phases) are introduced into the hidden states, and Gaussian mixture density is introduced to represent the complex conditioned observation density. The introduced sojourn state forms the semi-Markov structure, which naturally replicates the real transition of activity and gait during motion. Then, batch mode and on-line Expectation-Maximization (EM) algorithms are proposed, respectively, for model training and adaptive on-line recognition. The algorithm is tested on two datasets collected from wearable inertial sensors. The batch mode recognition accuracy reaches up to 95.16%, whereas the adaptive on-line recognition gradually obtains high accuracy after the time required for model updating. Experimental results show an improvement in performance compared to the other competitive algorithms.

## 1. Introduction

Locomotion activity has recently raised great research interest because of its significant potentials in many fields, e.g., rehabilitation for injured people [1], surveillance systems or health care for the elderly [2], daily activity management. Among these researches [3], many different types of sensors are used, such as camera, wireless beacon, electromyogram (EMG) sensors, electrocardiography (ECG) sensors, and inertial measurement units (IMUs). In a smart home, camera system or wireless beacon can help to understand the activity pattern of the host, and then provide suggestions for a healthy life or make a decision when an emergency is coming [4]. On the other hand, for the wearable sensors, EMGs can measure the electrical signal of muscles, while ECGs placed on specific body parts can monitor the heart rate. These kinds of signals can be used for evaluating the activity intensity. However, camera systems need to be pre-installed and calibrated, and they are also sensitive to the light. EMGs and ECGs have cables with the host, and they are sensitive to the moisture. By contrast, IMU sensors are small enough to be placed on the body and can be taken anywhere, providing information like 3D acceleration, angular rate, and magnetic field readings. In this work, given the advantages of using IMUs, we propose to use these sensors to collect the acceleration and angular rate of motion for the purpose of activity recognition.

Numerous single sensor-based and multiple sensors-based applications were developed under different scenarios. It seems that using multiple sensors is quite interesting and can help to recognize more complex activities. For example, Hsu et al. [5] utilized two IMU sensors placed on wrist and ankle to detect 10 daily activities and 11 sport activities. Xie et al. [6] used a hybrid system of inertial sensor and barometer to detect locomotion and static activities. In this paper, we are studying a generic model that can be applied to the recognition of lower limb locomotion activity, this kind of model can work for both single sensor-based and multiple sensors-based applications; the difference is that multiple sensors generate a higher observation dimension than a single sensor. For simplification, the proposed model will be validated through only one IMU sensor placed on the lower limb.

The work proposed here is, to some extent, the continuation of our previous work [7], where a non-parametric triplet Markov chain (TMC-HIST) was designed to detect four lower limb locomotion activities: walking, running, stair ascent and stair descent. TMC [8,9] is an extension of the hidden Markov chain model (HMC) that includes: the observation Y and hidden state X processes and a third auxiliary hidden state U process. While it keeps a similar parameter estimation and restoration algorithm as HMC, in the TMC-HIST, the hidden state process represented the considered activities, the auxiliary one modelized the gait cycle, and histograms were used to represent the non-Gaussian observation density conditioned on each hidden state. We also developed an adaptive on-line algorithm that is based on TMC-HIST to recognize the targeted activities. Results showed that the combination of lower limb activity and gait cycle can significantly improve the recognition performance, and the adaptive parameter updating can gradually fit the motion pattern of people. However, the non-parametric histogram represented the marginal density of observation along one sensor axis, it does not involve the correlation among the three axes of sensor. As a consequence, this weakness may cause a failure when recognizing the activity. In addition, the precision of histogram is highly dependent on the volume of data and the width of bins, which require large storage memory and will slow down the processing speed of on-line recognition.

In this work, in order to overcome the weaknesses of TMC-HIST, we focus on developing a new parametric TMC model that can recognize lower limb locomotion activities using one single IMU sensor. Besides, the proposed algorithm should be adaptive and on-line applicable as well, i.e., it can adjust its parameters at run-time to suit for the user. By introducing a sojourn hidden state process to form semi-Markov structure, it allows the hidden states X and U keep the same for a while, which is consistent with the activity and gait transition during the motion. Semi-Markov structure is embedded into the TMC to better mimic the real state transition properties. Multi-dimensional Gaussian mixture model (GMM) is introduced to represent the non-Gaussian conditioned observation densities, in the meantime, it involves the observation correlation among the sensor axes. With the introduction of semi-Markov structure and Gaussian mixture density, the specific TMC model will be referred to as SemiTMC-GMM in the remaining of this paper. Because of the parametric densities, an on-line parameter learning algorithm based on EM is applied. Therefore, our claimed contributions in this paper are:

- Semi-Markov structure is embedded into the TMC model to make the hidden state transition closer to the realistic motion.
- GMM is adopted to overcome the weakness of non-parametric density, while still allowing to model non-Gaussian data.
- An EM-based on-line learning algorithm is adopted to SemiTMC-GMM for making the algorithm work on-line.

The remaining of the paper is organized as follows. Section 2 depicts the state-of-the-art works in the field of activity recognition using wearable sensors. Section 3 gives the definition of conventional TMC model and gradually extends the model to SemiTMC-GMM. Then, how to apply the proposed model to recognize lower limb locomotion activities is presented in detail at the end of this Section. Section 4 depicts both batch mode and on-line mode parameter learning for the proposed model. In Section 5, the proposed recognition algorithm is tested on two datasets: one is the public dataset [10], and the other is our own dataset. In addition, the performance of the proposed algorithm is discussed compared to the competitive works. Finally, conclusions and future work are presented in the last Section.

## 2. Related Works

Numerous works have investigated human activity recognition (HAR) in the last decade. The methodologies used recently can generally be classified into two dominant categories: (i) traditional classifiers; (ii) deep learning methods.

For the first category, numerous classiffiers have been investigated. Parri et al. [11] proposed a fuzzy-logical classifier to identify lower limb locomotion mode, with the assistance of gait phases. The authors developed a lower limb wearable robot system that can help impaired people to perform locomotion activity. Chen et al. [12] proposed a robust activity recognition algorithm based on principal component analysis (PCA) and on-line support vector machine (OSVM), the algorithm obtained a robust recognition accuracy over a smartphone dataset collected in six different orientations. In the work [13], the authors compared the performances among the classifiers of SVM, Naive Bayes, k-Nearest Neighbour (kNN) and kStar. Results showed that kNN and kStar obtained the highest accuracy while Naive Bayes obtained the lowest. Zhao et al. [14] proposed a 2-layer model to detect six gait phases of walking, the algorithm used Neural Network (NN) to provide a pre-decision of gait phases to Hidden Markov Model (HMM), the final decision of gait phase from HMM obtained an accuracy of 98.11%. The limitation of this study is that only the activity of walking was considered, and the authors only tested their algorithm on straight forward walking, not free walking. In [15], hidden semi-Markov model (HSMM) and semi-Markov conditional random field (SMCRF) were applied to recognize human activity in smart home. The results showed that HSMM consistently outperformed HMM, while SMCRF obtained a similar result to CRF. However, because daily activities at home do not have stationary property, it is not practical to use a stationary transition matrix to represent the activity switches. Moreover, the authors only used Gaussian density to represent the conditioned observation density, which is quite limited to a complex scenario.

In the second category, deep learning-based methodologies are very prevalent. Generally, this kind of method is more inclined to image processing, so it needs to convert sensor data to image description to support extraction of discriminative features [16]. As reported in [17], convolutional neural network (CNN) is an important category of discriminative deep learning model for HAR. The work [18] proposed convolutional recurrent neural network to recognise daily activity; their algorithm gained an improvement of 6% compared to the state-of-the-art works. Recently, as reported in [19], transfer learning and semantic approach have raised great research interest. Bao [20] and Rokni [21] used transfer learning to automatically construct model for newly added wearable sensors; they obtained an accuracy enhancement between 9.3–10%. However, the recognition accuracy highly depends on the performance of labeling from source devices, thus it still requires a reliable method for recognition on a single sensor.

Some other methods can also be applied to dedicated applications and obtain good results. Schneider et al. [22] proposed an automatic extraction and selection method of highly relevant features, the method was tested on eight datasets and obtained a general accuracy over 90%. Rezaie et al. [23] proposed a feedback controller framework to adapt the sampling rate for better efficiency and higher accuracy. Dao et al. [24] introduced a man-in-loop decision architecture and data sharing among users and gradually obtained a high accuracy.

In fact, people perform lower limb locomotion activities every day, such as moving from one place to another place and doing sports like running and cycling. … There are a lot of methods that have been proposed for HAR, while to our best knowledge, very few methods can be found that are specially designed for lower limb locomotion activities, including but not limited to activities like walking and jogging [25].

## 3. Model

In this section, the conventional TMC model is firstly introduced, then it is gradually equipped with more sophisticated structures, i.e., applying Gaussian mixture to TMC to obtain the TMC-GMM model and then applying semi-Markov structure to TMC-GMM to obtain the SemiTMC-GMM model. Afterward, a detailed description of on-line EM algorithm suited for SemiTMC-GMM is given. As a matter of fact, these additional processes can be naturally added because of the high generality of the TMC model through the flexibility of the auxiliary processes.

### 3.1. Triplet Markov Chain

Consider two discrete stochastic processes $X=(X_{1},\ldots ,X_{N})$ and $U=(U_{1},\ldots ,U_{N})$ as hidden states, where $X_{n}\in \Lambda =\left\{1,\ldots ,r\right\}$ and $U_{n}\in \Gamma =\left\{1,\ldots ,\tau \right\}$, n∈{1,…,N}. Let $Y=(Y_{1},\ldots ,Y_{N})$ be a real-valued process representing the observation of the model, each $Y_{n}\in R^{w}$, where *w* is the observation dimension. In this paper, the hidden state X refers to the activity to be recognized, U refers to the introduced gait or leg cycle, while the observation Y is the features extracted from sensor readings. The details of how to apply the model to recognize lower limb locomotion activity are described in Section 3.4. Then, the triplet T=(V,Y), with V=(X,U) is a TMC if T is Markovian. It should be noted here that, in classic TMC, none of processes X,U,Y,(X,U),(X,Y),(U,Y) are necessarily Markovian.

Let the realizations of $X_{n}$, $U_{n}$ and $Y_{n}$ be denoted by their lower cases $x_{n}$, $u_{n}$, and $y_{n}$, respectively, so $v_{n}=\left(x_{n},u_{n}\right)$, $t_{n}=\left(v_{n},y_{n}\right)$. In addition, for simplification, we will denote the probabilities $p(X_{n}=x_{n},U_{n}=u_{n}|Y_{1}=y_{1},\ldots ,Y_{N}=y_{N})$ by $p(x_{n},u_{n}|y_{1}^{N})$, $p(X_{n}=x_{n},U_{n}=u_{n}|Y_{1}=y_{1},\ldots ,Y_{n}=y_{n})$ by $p(x_{n},u_{n}|y_{1}^{n})$, for example. In TMC transitions form $p(t_{n+1}|t_{n})$ can be expressed in different forms, let us consider the following one:

(1) $$p\left(t_{n+1}|t_{n}\right)=p\left(v_{n+1}|v_{n},y_{n}\right)p\left(y_{n+1}|v_{n+1},v_{n},y_{n}\right).$$

**Figure 1:**
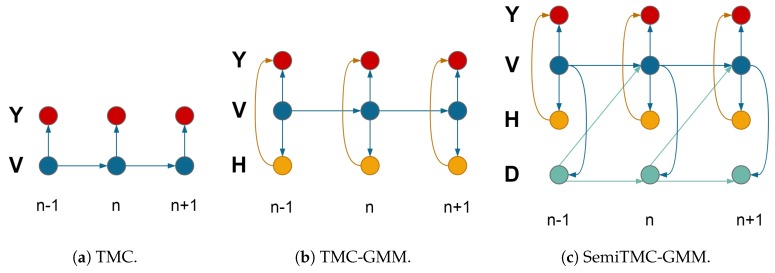
Dependency graphs. TMC = triplet Markov chain; GMM = Gaussian mixture model.

In the application of this paper we will assume that $p\left(v_{n+1}|v_{n},y_{n}\right)=p\left(v_{n+1}|v_{n}\right)$ and $p\left(y_{n+1}|v_{n+1},v_{n},y_{n}\right)=p\left(y_{n+1}|v_{n+1}\right)$. So the transition is simplified to

(2) $$p\left(t_{n+1}|t_{n}\right)=p\left(v_{n+1}|v_{n}\right)p\left(y_{n+1}|v_{n+1}\right),$$

which provides process T with the structure of a classical HMC. For simplification, this simplified TMC is referred to as TMC in the remaining. The first term $p\left(v_{n+1}|v_{n}\right)$ in Equation (2) is the state transition probability, the dimension of the matrix is (r×τ)×(r×τ). The second term is the probability of observing $y_{n}$ conditionally to each state. Most of the time, this kind of density is modeled by Gaussian distributions:

(3) $$p\left(y_{n}|v_{n}=i\right)∼N\left(\mu_{i},\Sigma_{i}\right),i\in \Lambda \times \Gamma ,$$

where $\mu_{i}$ and $\Sigma_{i}$ are the mean and variance. The dependency graph of this particular TMC is shown in Figure 1a. Regardless of the probabilistic links inside the nodes related to V, the dependency of Y and V is just in the form of HMC.

For obtaining the probability of individual $x_{n}$ and $u_{n}$ conditioned on $y_{1}^{n}$, $y_{1}^{N}$, we only need to compute the marginal probability of $p(x_{n},u_{n}|y_{1}^{n})$ and $p(x_{n},u_{n}|y_{1}^{N})$. Indeed, we have

(4) $$\begin{array}{cc} p(x_{n}|y_{1}^{n}) & =\sum_{u_{n}}p\left(x_{n},u_{n}|y_{1}^{n}\right),\\ p(x_{n}|y_{1}^{N}) & =\sum_{u_{n}}p\left(x_{n},u_{n}|y_{1}^{N}\right). \end{array}$$

Likewise, $p(u_{n}|y_{1}^{n})$ and $p(u_{n}|y_{1}^{N})$ can be obtained in a similar way. $p(x_{n},u_{n}|y_{1}^{n})$ and $p(x_{n},u_{n}|y_{1}^{N})$ are the probabilities if known the observation $y_{1}^{n}$ and $y_{1}^{N}$, commonly they are called filtering probability and smoothing probability, respectively. Similar meaning can be deduced for $p(x_{n}|y_{1}^{n})$, $p(x_{n}|y_{1}^{N})$, $p(u_{n}|y_{1}^{n})$, $p(u_{n}|y_{1}^{N})$. Then, the estimated hidden state will be obtained via MPM (Maximum Posterior Mode) criterion using the smoothing probability:

(5) $$\begin{array}{cc} {\hat{x}}_{n} & =\arg\;\max_{x_{n}\in \Lambda }p\left(x_{n}|y_{1}^{N}\right),\\ {\hat{u}}_{n} & =\arg\;\max_{u_{n}\in \Gamma }p\left(u_{n}|y_{1}^{N}\right). \end{array}$$

### 3.2. TMC Embedding a Gaussian Mixture Model

When extending TMC to TMC-GMM, it needs to introduce Gaussian mixture density into the conditioned observation probability. In fact, embedding GMM in TMC can be regarded as introducing a new statistic process $H=(H_{1},\ldots ,H_{N})$ into TMC, where $H_{n}$ takes its value $h_{n}$ in a finite set K={1,…,κ} and κ is the number of Gaussian components in the mixture. Please remind that H has no realistic meaning, it is just a latent variable in the model to introduce the mixtures. Let $c_{ij}$ be the weight of *j*th Gaussian mixture component when $v_{n}=i$, with the constraint $\sum_{j=1}^{\kappa }c_{ij}=1$. $\mu_{ij}$ and $\Sigma_{ij}$ are the mean value and covariance of the Gaussian mixture component. Denoting Z=(T,H) and assuming $p\left(h_{1}^{N}|v_{1}^{N}\right)=\prod_{n=1}^{N}p\left(h_{n}|v_{n}\right)$, Z is Markovian with transitions:

(6) $$p\left(z_{n+1}|z_{n}\right)=p\left(v_{n+1}|v_{n}\right)p\left(h_{n+1}|v_{n+1}\right)p\left(y_{n+1}|v_{n+1},h_{n+1}\right),$$

where

(7a) $$p\left(y_{n}|v_{n}=i,h_{n}=j\right)∼N\left(\mu_{ij},\Sigma_{ij}\right),\;i\in \Lambda \times \Gamma ,j\in K,$$

(7b) $$p\left(y_{n}|v_{n}\right)=\sum_{j=1}^{\kappa }c_{ij}\cdot p\left(y_{n}|v_{n}=i,h_{n}=j\right),$$

with $p\left(h_{n}=j|v_{n}=i\right)=c_{ij}$. We can see that Equations (6) and (7a) are extensions of Equations (2) and (3), by introducing a new process H. The dependency graph of TMC-GMM is shown in Figure 1b.

Please notice that the only difference between TMC and TMC-GMM is the Gaussian densities in TMC are replaced with Gaussian mixtures, all the other calculations remain the same. Then estimating the individual $x_{n}$ and $u_{n}$ in TMC-GMM follows the same as in TMC, by using Equations (4) and (5).

### 3.3. Semi TMC-GMM

In the Markov model considered in our previous work [7], the remaining time of the sojourn of the hidden state $v_{n}$ is of geometric distribution. While considering V as semi-Markovian seems to better suited to our problematic, as in general, $v_{n}$ has no geometric remaining sojourn time. For example, the gait phase has a minimum duration, while in geometric distribution the maximal probability is for null duration. In real applications of classic hidden semi-Markov model (HSMM) [26,27], there is a fixed maximum sojourn time for each possible value of $v_{n}$. When $v_{n}$ switches to a new value, the maximal possible random sojourn time is shorter than a fixed value *M*. Once the sojourn time has elapsed at time *n*, the hidden state must change to a different value, i.e., $p(v_{n+1}=v_{n})=0$. This implies that the maximum sojourn time should be large enough to cover the largest possible sojourn time, which appears as a drawback in our application. In another semi-Markov approach described in [28] that we adopt here, the random sojourn time (just after having switched) is not the exact duration of the state, but the minimum sojourn time. This means that once the sojourn time elapsed, the next hidden state is possible to stay the same. This character allows make the maximum value *M* significantly smaller than the one in classic HSMM, which accelerates the entire method since the dimension of transition matrix is reduced.

To be more precise, consider a new stochastic process $D=(D_{1},\ldots ,D_{N})$ that represents the minimum remaining sojourn time in a given hidden state $v_{n}$, and the possible realization of each $D_{n}$ (denoted by $d_{n}$) takes its value in L={0,1,…,ℓ}. Thus for $V_{n}=v_{n}$ and $D_{n}=d_{n}$, we have $v_{n}=v_{n+1}=\ldots =v_{n+d_{n}}$. And $v_{n+d_{n}}$ is obtained *w.r.t.* $p(v_{n+d_{n}+1}|v_{n+d_{n}})$, which is a transition similar to the ones in the TMC and TMC-GMM. Thus, $v_{n+d_{n}+1}$ is possible to be the same as $v_{n+d_{n}}$. Once $v_{n+1}$ is set, a new minimum sojourn time $d_{n+1}$ is obtained in L={0,1,…,ℓ}. Please notice that for $D_{n}=d_{n}\neq 0$, there is $D_{n+1}=d_{n+1}=d_{n}-1$, which is specified in Equation (10).

Finally, SemiTMC-GMM is extended from TMC-GMM Z via the couple (Z,D), which follows the transition probabilities:

(8) $$p\left(z_{n+1},d_{n+1}|z_{n},d_{n}\right)=p\left(v_{n+1}|z_{n},d_{n}\right)p\left(h_{n+1}|v_{n+1}\right)p\left(d_{n+1}|v_{n+1},d_{n}\right)p\left(y_{n+1}|v_{n+1},h_{n+1}\right),$$

(9) $$p\left(v_{n+1}|z_{n},d_{n}\right)=\left\{\begin{array}{cc} \delta_{v_{n}}\left(v_{n+1}\right), & d_{n}>0\\ p^{*}\left(v_{n+1}|v_{n}\right), & d_{n}=0 \end{array}\right.,$$

(10) $$p\left(d_{n+1}|v_{n+1},d_{n}\right)=\left\{\begin{array}{cc} \delta_{d_{n}-1}\left(d_{n+1}\right), & d_{n}>0\\ p(d_{n+1}|v_{n+1}), & d_{n}=0 \end{array}\right.,$$

where δ is the Kronecker function ($\delta_{a}\left(b\right)=1$ for a=b and $\delta_{a}\left(b\right)=0$ for a≠b).

The properties of the four terms on the right side of Equation (8) are clarified in the following:

1. In Equation (9), when $d_{n}=0$, the transition $p^{*}\left(v_{n+1}|v_{n}\right)$ behaves the same as the state transition of TMC and TMC-GMM, which means that $v_{n+1}$ can be different from or same as $v_{n}$, depending on the distribution of $p^{*}\left(v_{n+1}|v_{n}\right)$.
2. $p(d_{n+1}|v_{n+1},d_{n})$ is the probability of the minimal remaining sojourn time of $v_{n+1}$, conditioned on $v_{n+1}$ and $d_{n}$.
3. $p(h_{n+1}|v_{n+1})$ and $p(y_{n+1}|v_{n+1},h_{n+1})$ are same as the ones in TMC-GMM, shown in Equation (7a).

Now, the Equations (9) and (10) together describe how the hidden states, $V_{n}$ and $D_{n}$, transfer in SemiTMC-GMM.

The dependency graphs of the three models, i.e., TMC, TMC-GMM, and SemiTMC-GMM, are shown in Figure 1. The couple V=(X,U) is regarded as one hidden state for reducing the complexity of the graphs, as well as reminding that the total number of processes involved in the three models are 3, 4, and 5, respectively.

Estimating the individual $x_{n}$ and $u_{n}$ is different from both TMC and TMC-GMM, for the sense of introducing the sojourn state $D_{n}$. The probabilities of $x_{n}$ can be obtained by

(11) $$\begin{array}{cc} p(x_{n}|y_{1}^{n}) & =\sum_{u_{n}}\sum_{d_{n}}p\left(x_{n},u_{n},d_{n}|y_{1}^{n}\right),\\ p(x_{n}|y_{1}^{N}) & =\sum_{u_{n}}\sum_{d_{n}}p\left(x_{n},u_{n},d_{n}|y_{1}^{N}\right). \end{array}$$

The probabilities $p(x_{n},u_{n},d_{n}|y_{1}^{n})$ and $p(x_{n},u_{n},d_{n}|y_{1}^{N})$ are the filtering and smoothing probability of the hidden state in SemiTMC-GMM, respectively. Likewise, the probabilities $p(u_{n}|y_{1}^{n})$ and $p(u_{n}|y_{1}^{N})$ are obtained in a similar way. Finally, the estimated hidden state ${\hat{x}}_{n}$ and ${\hat{u}}_{n}$ can be obtained by Equation (5).

To summarize, the proposed SemiTMC-GMM is a model contains five stochastic processes X, U, D, H, Y, with Markov distribution of $Z^{*}=\left(X,U,D,H,Y\right)$. The process X models the activities we are looking for, Y models the observation, U models the introduced gait or leg phase, D models the semi-Markovianity of V=(X,U), and H models the presence of Gaussian mixtures. Thus, $Z^{*}=\left(V,W,Y\right)$ can be regarded as a classic TMC with hidden state V, observed Y, and an additional latent W=(D,H).

### 3.4. Application of SemiTMC-GMM

The question is now how to apply the proposed model to recognise lower limb locomotion activities. In our previous work [7], gait cycle was introduced into the estimation of four locomotion activities, and the results show that it can improve the accuracy. As introduced in [29], one gait cycle can be divided into four gait phases, i.e., stance, push-up, swing, and step down. In this work, we are pursuing a method that does not require the sensor to be placed on the feet only. On the contrary, it can be placed on different places of the lower limb, such as thigh, shank, or foot. The segmentation of gait cycle is based on the motion of foot, so similarly we can define ‘leg cycle’ based on the motion of leg. One leg cycle can be segmented into four leg phases, which are low position, lifting, high position, and dropping.

**Figure 2:**
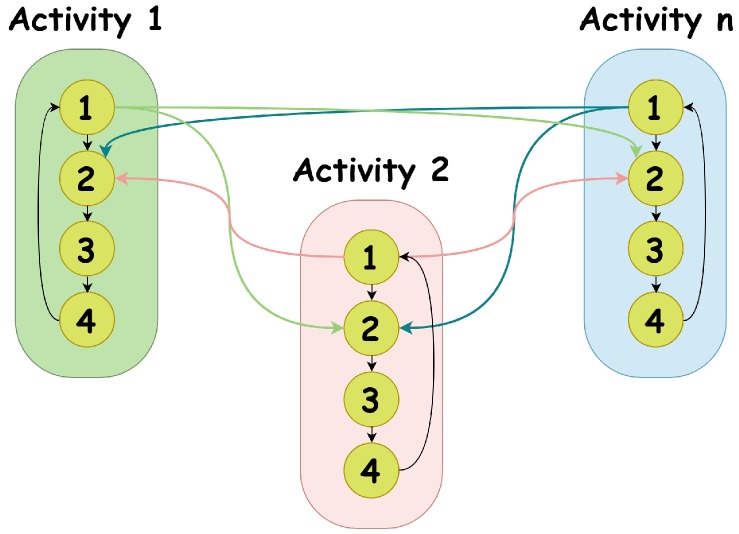
Hidden state transition graph. The activities represent *X*, and the numbers 1–4 represent *U* and stand for the four gait phases, or leg phases.

Let assume the hidden state *X* represents the activity, and *U* be the gait cycle or leg cycle. Thus, the dimension of Λ (*r*) depends on the number of activities; while for Γ, τ is equal to 4. The transition of *X* and *U* follows a specific order because the feet move from attaching on the ground to swinging in the air alternately, or the legs switch between lifting to dropping. Therefore, we define a specific transition graph for *X* and *U*. As shown in Figure 2, the numbers 1–4 represent the hidden state *U*, the four gait and leg phases. We can see that *U* transfers from phase 1 to phase 4 and back to phase 1 again cyclically if the activity does not change. While the activity is switching, *U* transfers from phase 1 of the previous activity to phase 2 of the current activity.

The hidden states *H* and *D* are not the final goals of the recognition, and they have no physical meaning neither. For simplification, the dimension of *L* (*ℓ*) is set to 9. This value was determined by our experience, a too small value will make the results of SemiTMC-GMM no difference from that of TMC-GMM, while a too large value will cost too much time for running the code. The performance of different GMM components number (κ) is evaluated on two datasets, as depicted in Section 5.

The observation Y is the feature extracted from the sensor readings. The utilized features are the sliding mean value and standard deviation, by calculating the mean value and standard deviation of acceleration and angular rate within a sliding window length. Since IMUs measure 3-dimensional acceleration and angular rate, then the dimension of the observation Y (*w*) equals to 12. The initialization of the hidden states is the same as the one in our previous work [7], so it will not be repeated here. Afterward, based on the initial hidden states $v_{1}^{N}$ and features $y_{1}^{N}$, the initial conditioned GMM density $p(y_{n}|v_{n},h_{n})$ can be easily obtained if the mixture number κ is known. When the initialization is done, batch mode EM algorithm can be applied to train the model, which will be described in details in Section 4. Then, the trained model can be used for the batch mode testing, or, as the initial model of on-line EM algorithm.

## 4. Parameter Estimation

From the previous section, it is now clear how the hidden state transfers and how to compute the observation probability. In this section, we focus on how to obtain the filtering and smoothing probabilities, and to apply parameter updating based on the on-line EM algorithm.

Before starting the explanation, we need to introduce the parameter set first. As described in the previous Section, the parameter set can be defined as $\theta =\{\zeta_{k},a_{lk},c_{ij},\mu_{ij},\Sigma_{ij}\}$, in which $\zeta_{k}$ is the initial probability of hidden state, and $a_{lk}$ is the *l*-th row and *k*-th column element in the transition matrix *A*. Because GMM density only depends on $v_{n}$, then i∈Λ×Γ, j∈K. In SemiTMC-GMM, the entire hidden state is (V,D), and then l,k∈Λ×Γ×L, and l,k is equal to the couple of $\left(i,d_{n}\right)$. Therefore, the initial probability becomes $\zeta_{k}=p\left(\left(v_{1},d_{1}\right)=k\right)$, and $a_{lk}=p\left(\left(v_{n+1},d_{n+1}\right)=k|\left(v_{n},d_{n}\right)=l\right)$. For simplification, the indices i,j,l,k will keep the same meaning and will no longer be specified in the remaining.

### 4.1. Batch Mode EM Algorithm

The batch mode parameter restoration using EM algorithm is quite simple and has been utilized in many researches. A dominated way to do this is using the well-known Baum-Welch algorithm. This is an algorithm that makes the expectation step and maximization step recursively. Here we simply describe how to extend the expectation and maximization steps to SemiTMC-GMM model, within one iteration of the EM algorithm. A simple recall of the forward and backward calculations [7] are displayed below:

(12) $$\begin{array}{cc} \alpha_{1}\left(k\right) & =p\left(\left(v_{1},d_{1}\right)=k,y_{1}\right),\\ \alpha_{n}\left(k\right) & =\sum_{(v_{n-1},d_{n-1})\in \Lambda \times \Gamma \times L}\alpha_{n-1}\left(k\right)\cdot p\left(\left(v_{n},d_{n}\right)|\left(v_{n-1},d_{n-1}\right)\right)\cdot p\left(y_{n}|\left(v_{n},d_{n}\right)\right),\\ \beta_{N}\left(k\right) & =1,\\ \beta_{n}\left(k\right) & =\sum_{(v_{n+1},d_{n+1})\in \Lambda \times \Gamma \times L}p\left(\left(v_{n+1},d_{n+1}\right)|\left(v_{n},d_{n}\right)\right)\cdot p\left(y_{n+1}|\left(v_{n+1},d_{n+1}\right)\right)\cdot \beta_{n+1}\left(k\right). \end{array}$$

In the above equations, the $\alpha_{n}\left(k\right)$ and $\beta_{n}\left(k\right)$ are the forward and backward calculations, $p\left(\left(v_{n},d_{n}\right)|\left(v_{n-1},d_{n-1}\right)\right)$ is the state transition probability that has been described in Equations (8)–(10), $p\left(y_{n}|\left(v_{n},d_{n}\right)\right)=p\left(h_{n}|v_{n}\right)p\left(y_{n}|v_{n},h_{n}\right)$ is the conditioned observation density.

Then, the algorithm requires the following probabilities:

(13) $$\gamma_{n}\left(k\right)=p\left(\left(v_{n},d_{n}\right)=k|y_{1}^{N}\right)=\frac{\alpha_{n}\left(k\right)\beta_{n}\left(k\right)}{\sum_{k^{\prime }\in \Lambda \times \Gamma \times L}\alpha_{n}\left(k^{\prime }\right)\beta_{n}\left(k^{\prime }\right)},$$

(14) $${\tilde{\gamma }}_{n}\left(i\right)=\sum_{d_{n}}\gamma_{n}\left(\left(i,d_{n}\right)\right)=\sum_{d_{n}}p\left(v_{n}=i,d_{n}|y_{1}^{N}\right),$$

(15) $${\tilde{\gamma }}_{n}\left(i,j\right)=\tilde{\gamma }\left(i\right)\cdot \frac{c_{ij}p\left(y_{n}|v_{n}=i,h_{n}=j\right)}{\sum_{j^{\prime }\in K}c_{ij^{\prime }}p\left(y_{n}|v_{n}=i,h_{n}=j^{\prime }\right)},$$

(16) $$\xi_{n}\left(l,k\right)=\frac{\alpha_{n}\left(l\right)\cdot p\left(y_{n+1},h_{n+1},\left(v_{n+1},d_{n+1}\right)=k|y_{n},h_{n},\left(v_{n},d_{n}\right)=l\right)\cdot \beta_{n+1}\left(k\right)}{\sum_{l^{\prime },k^{\prime }\in \Lambda \times \Gamma \times L}\left\{\alpha_{n}\left(l^{\prime }\right)\cdot p\left(y_{n+1},h_{n+1},\left(v_{n+1},d_{n+1}\right)=k^{\prime }|y_{n},h_{n},\left(v_{n},d_{n}\right)=l^{\prime }\right)\cdot \beta_{n+1}\left(k^{\prime }\right)\right\}}.$$

$\gamma_{n}\left(k\right)$ is the probability of $\left(v_{n},d_{n}\right)$ conditioned on all observed data $y_{1}^{N}$. ${\tilde{\gamma }}_{n}\left(k\right)$ is the marginal probability of $\gamma_{n}\left(k\right)$ over $d_{n}$, this probability is the one that we are looking for to estimate the concerning hidden state $v_{n}$. ${\tilde{\gamma }}_{n}\left(i,j\right)$ is the probability of each Gaussian component *w.r.t.* ${\tilde{\gamma }}_{n}\left(k\right)$; this probability helps to compute the parameters related to Gaussian mixture, i.e., $c_{kj}$, $\mu_{kj}$, $\Sigma_{kj}$. $\xi_{n}\left(l,k\right)$ is the joint probability of $(v_{n},d_{n})=l$ and $(v_{n+1},d_{n+1})=k$ conditioned on $y_{1}^{N}$. Here we give the formula of parameter update by using Equations (13)–(16):

(17) $$\zeta_{k}=\gamma_{1}\left(k\right),$$

(18) $$a_{lk}=\frac{\sum_{n=1}^{N-1}\xi_{n}\left(l,k\right)}{\sum_{n=1}^{N-1}\gamma_{n}\left(l\right)},$$

(19) $$c_{ij}=\frac{\sum_{n=1}^{N}{\tilde{\gamma }}_{n}\left(i,j\right)}{\sum_{n=1}^{N}{\tilde{\gamma }}_{n}\left(i\right)},$$

(20) $$\mu_{ij}=\frac{\sum_{n=1}^{N}{\tilde{\gamma }}_{n}\left(i,j\right)y_{n}}{\sum_{n=1}^{N}{\tilde{\gamma }}_{n}\left(i,j\right)},$$

(21) $$\Sigma_{ij}=\frac{\sum_{n=1}^{N}{\tilde{\gamma }}_{n}\left(i,j\right){\left(y_{n}-\mu_{ij}\right)}^{T}\left(y_{n}-\mu_{ij}\right)}{\sum_{n=1}^{N}{\tilde{\gamma }}_{n}\left(i,j\right)}.$$

In fact, Equations (13)–(16) are the expectation step in one iteration of EM algorithm, while Equations (17)–(21) are the maximization step. Then, the parameter can be learned by recursively performing the two steps until the iteration number exceeds a pre-defined value, maximally 100 iterations, for example.

### 4.2. Sufficient Data Statistics

Since Gaussian Markov models belong to the exponential family, the likelihood function of SemiTMC-GMM can be written in the form of [30]

(22) $$p_{\theta }\left(z_{n},d_{n}\right)=f\left(z_{n},d_{n}\right)\exp\left(\langle s\left(z_{n},d_{n}\right),\psi \left(\theta \right)\rangle -J\left(\theta \right)\right),$$

where $s(z_{n},d_{n})$ is a vector of complete-data sufficient statistics belonging to convex set *S*, 〈·,·〉 denotes the scalar product, function ψ(·) maps θ to the natural parametrization, and J(·) is the log-partition function. For SemiTMC-GMM, the definition of statistics is

(23) $$\begin{array}{cc} s_{n^{\prime },lk}^{\left(1\right)} & =𝟙\{\left(v_{n^{\prime }},d_{n^{\prime }}\right)=l,\left(v_{n^{\prime }+1},d_{n^{\prime }+1}\right)=k\}, \end{array}$$

(24) $$\begin{array}{cc} s_{n^{\prime },k}^{\left(2\right)} & =𝟙\{\left(v_{n^{\prime }},d_{n^{\prime }}\right)=k\}, \end{array}$$

(25) $$\begin{array}{cc} s_{n^{\prime },ij}^{\left(3\right)} & =𝟙\{v_{n^{\prime }}=i,h_{n^{\prime }}=j\}, \end{array}$$

(26) $$\begin{array}{cc} s_{n^{\prime },ij}^{\left(4\right)} & =𝟙\left\{v_{n^{\prime }}=i,h_{n^{\prime }}=j\right\}y_{n^{\prime }}, \end{array}$$

(27) $$\begin{array}{cc} s_{n^{\prime },ij}^{\left(5\right)} & =𝟙\left\{v_{n^{\prime }}=i,h_{n^{\prime }}=j\right\}y_{n^{\prime }}^{T}y_{n^{\prime }}, \end{array}$$

where 𝟙{·} is the indicator function, $n^{\prime }=1,\ldots ,N$. Then, the statistics vector at time $n^{\prime }$ is of the form $s_{n^{\prime }}=\left\{s_{n^{\prime },lk}^{\left(1\right)},s_{n^{\prime },k}^{\left(2\right)},s_{n^{\prime },ij}^{\left(3\right)},s_{n^{\prime },ij}^{\left(4\right)},s_{n^{\prime },ij}^{\left(5\right)}\right\}$. Consequently, the sufficient statistics $S_{n}$ is the expectation of $s_{n^{\prime }}$ conditioned on $y_{1}^{n}$:

(28) $$S_{n}=\frac{1}{n}E_{\theta }\left(\sum_{n^{\prime }=1}^{n}s_{n^{\prime }}\right)|y_{1}^{n}.$$

Denote $S_{n}=\left\{S_{n,lk}^{\left(1\right)},S_{n,k}^{\left(2\right)},S_{n,ij}^{\left(3\right)},S_{n,ij}^{\left(4\right)},S_{n,ij}^{\left(5\right)}\right\}$, in which the elements are the expectation of the ones with respect to $s_{n^{\prime }}$. Now, comparing the Equation groups (13)–(21) and (23)–(28), we can reform the parameter update Equations (17)–(21) with sufficient statistics:

(29) $${\tilde{S}}_{n,i}^{\left(2\right)}=\sum_{d_{n}}S_{n,(i,d_{n})}^{\left(2\right)},$$

(30) $$\zeta_{k}=S_{1,k}^{\left(2\right)},$$

(31) $$a_{n,lk}=S_{n,lk}^{\left(1\right)}/S_{n,k}^{\left(2\right)},$$

(32) $$c_{n,ij}=S_{n,ij}^{\left(3\right)}/{\tilde{S}}_{n,i}^{\left(2\right)},$$

(33) $$\mu_{n,ij}=S_{n,ij}^{\left(4\right)}/S_{n,ij}^{\left(3\right)},$$

(34) $$\Sigma_{n,ij}=S_{n,ij}^{\left(5\right)}/S_{n,ij}^{\left(3\right)}-\mu_{n,ij}^{T}\mu_{n,ij},$$

in which $a_{n,lk}$, $c_{n,ij}$, $\mu_{n,ij}$, $\Sigma_{n,ij}$ are the updated parameters at time *n*.

**Remark** **1.** *Replacing n with N in Equation (28), which means all the observed data $y_{1}^{N}$ are used, $S_{N}$ is then called as complete sufficient statics. Therefore, using $S_{N}$ to compute the parameters in Equations (30)–(34) will be exactly the batch mode parameter learning that is given in Equations (17)–(21).*

### 4.3. On-Line Estimation

In the previous section, we have discussed about how to use sufficient statistics to learn θ in batch mode. In order to apply the on-line estimation, a common way [30] is to update the sufficient statistics when a new observed data come in:

(35) $$S_{n+1}=\left(1-\rho_{n+1}\right)\cdot S_{n}+\rho_{n+1}\cdot E_{\theta_{n}}\left(s_{n+1}|y_{n+1}\right),$$

where $\rho_{n}$ is the stepsize sequence that satisfies $\sum_{n=1}^{\infty }\rho_{n}=\infty$, $\sum_{n=1}^{\infty }\rho_{n}^{2}<\infty$. Normally it is set to $\rho_{n}=1/n$. Then, the new parameter $\theta_{n+1}$ is available by Equations (29)–(34). The estimation of $x_{n+1}$, $u_{n+1}$ can be obtained by Equations (5) and (11).

In this paper, we do not update θ at every sampling time. Instead, we set a window length $W_{l}$ and accumulate the latest $W_{l}$ observed data first. Then use Equations (13)–(16) to get the smoothed result, compute the sequenced statistics $s_{n}{|}_{1}^{W_{l}}$ for all the $W_{l}$ data by Equations (23)–(27). Afterward, update the sequenced sufficient statistics $S_{n}{|}_{1}^{W_{l}}$ and $\theta_{n}{|}_{1}^{W_{l}}$ by Equations (35) and (29)–(34), respectively. It should be noticed that in on-line mode, the initial probability $\zeta_{k}$ is not necessary.

**Figure 3:**
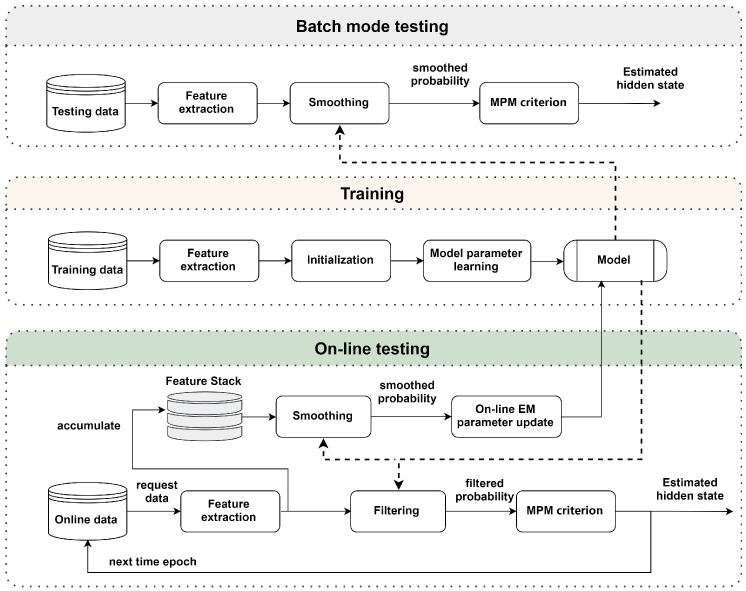
Diagram of the training stage, and the testing stage for both batch mode and on-line testing.

After describing the batch mode and on-line parameter learning, a diagram of the training and testing is displayed in Figure 3. In the training stage, the block of model parameter learning is the Baum-Welch algorithm. The trained model is used in both the two kinds of testing, the model is updated in on-line testing, but not in the batch mode testing. Besides, the estimated hidden state of batch mode is from the smoothed probability, whereas the one of on-line is from the filtered probability.

## 5. Experimental Results

Two datasets are used to validate the proposed algorithm. The first dataset is the Sports and Daily Activities (SDA) dataset [10], in which eight subjects were enrolled to perform 19 daily and sports activities while wearing five Xsens MTx (Details of Xsens MTx can be found in http://www.xsens.com/en/general/mtx)) IMUs on their torso, left arm, right arm, left thigh, and right thigh, all the sensors measure the acceleration and angular rate of the body parts where the sensors are placed. The five sensor placements are determined in this dataset because the involved activities are not only lower limb locomotion activities but also include static activities and upper limb activities, such as sitting, lying, rowing, and playing basketball… So they used the 5 sensors to collect the motion data from different parts of the body. However, in this paper, the proposed algorithm is designed for recognizing the lower limb locomotion activity with periodic gait or leg cycle performed by healthy people. Since we only care about the movement of the lower limbs and healthy people have a symmetric motion of the two legs, then it is possible to use only one sensor placed on either the left or right leg to recognize the considered activities. Therefore, we only use the sensor placed on the right thigh to validate our algorithm. The sensor sampling rate was set to 25 Hz, the acceleration sensing range was set to ±18 g, the angular rate sensing range was set to ±1200°/s. Because the objective of the proposed algorithm is to detect lower limb locomotion activities that have gait cycle or leg cycle, while the 19 activities consists of both lower limb locomotion activities with and without the cycles, then only 11 suitable activities out of the total are selected in this work: walk in parking lot, walk on treadmill with incline, walk on treadmill on flat, stair descent, stair ascent, run on treadmill, jump, exercise on stepper, exercise of cycling in vertical position, exercise of cycling in horizontal position, exercise on cross trainer. These 11 locomotion activities of SDA dataset are referred to as D1A1 to D1A11 in the remaining of this paper. In the dataset, the subjects performed each activity for about 5 min separately, and each activity was divided into 60 segments of 5 s. Therefore, there are 60 × 8 = 480 segments for each activity. In order to make the dataset available for our algorithm, we firstly combined the 60 segments of one activity from one subject to recover the 5 min activity, then combined the data of the same activity from all the subjects to form 40 min for each activity, the final data was obtained by combining the 11 activities. Thus, the duration of the data is 11 × 40 = 440 min.

There are only 7500 samplings for each experiment of SDA, and the data length is not long enough to use on-line EM recognition. Therefore, we utilize the second dataset for the validation of the proposed on-line EM algorithm. This second dataset, described in [7], is called Locomotion of Foot-mounted IMU (LMFIMU) dataset (the dataset and its details are available on the website: https://github.com/unilee/TMC_LowerLimbActs). Ten subjects were enrolled to perform a specific experiment that lasts nearly 30 min with a Shimmer3 (Shimmer3 GSR+, details at the manufacturer’s site http://www.shimmersensing.com/images/uploads/docs/ConsensysPRO_Spec_Sheet_v1.1.0.pdf) IMU mounted on the right shoe. The sensor is placed on the shoe in LMFIMU but not the thigh as in SDA dataset is because both feet and thighs have a periodic pattern when people are performing the lower limb locomotion activities. In fact, the sensor can be placed at anywhere of the lower limbs in this study. So foot is chosen in the LMFIMU dataset to show that the proposed algorithm is not restricted to only one sensor position. Because of the enrolled 10 healthy subjects, left foot and right foot have the same behavior, then the sensor is placed on the right shoe of each subject. Each subject was asked to perform one experiment, which contained two identical sections of a sequence of four locomotion activities: walking, running, stair ascent and stair descent. Therefore, the performance of the second section will be improved compared to the first section, if the on-line algorithm can gradually learn the activity pattern of the subject. The four locomotion activities are referred to as D2A1 to D2A4 in the rest of this paper. The sensor sampling rate was set to 100 Hz, so the data length is long enough for the on-line EM algorithm. The sensing range of the acceleration and angular rate are ±8 g and ±1000°/s, respectively.

The proposed SemiTMC-GMM model is compared with TMC-GMM to see the advancement of semi-Markov structure in recognizing lower limb locomotion activities. GMM is implemented by different κ to see the impact of the GMM components number that has on recognition accuracy.

### 5.1. SDA Dataset

The batch mode recognition is tested by a leave-one-out cross-validation (LOOCV) strategy, i.e., taking one subject for testing and the others for training, then make the test for all the subjects. The sliding window length of feature extraction is set to 5. The length is set based on our experience; normally it depends on the shortest gait or leg phase of the activities, and a window length larger than the shortest duration may conceal some information of the phase, while too small length may cause a badly calculated standard deviation. Both the SemiTMC-GMM and TMC-GMM model are involved in the validation, and the GMM mixture number κ is set to 1, 3, 6, and 9, respectively. Particularly when κ=1, the conditioned observation density yields to the conventional Gaussian distribution.

**Figure 4:**
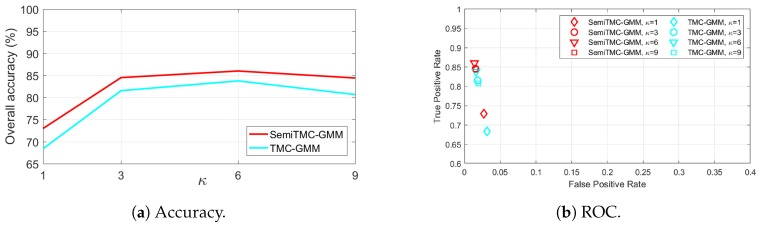
The batch mode recognition performance of the Sports and Daily Activities (SDA) dataset, of the SemiTMC-GMM, and TMC-GMM models, according to different GMM mixture number *κ*. ROC = Receiver Operating Characteristics.

The overall accuracy of batch mode recognition on SDA dataset is shown in Figure 4a. As it can be seen, SemiTMC-GMM achieves an accuracy improvement of about 2–3% compared to TMC-GMM. The proposed model reaches the highest accuracy of 86.00% when κ=6, while the one of TMC-GMM is 83.76%. Meanwhile, TMC-HIST obtains the lowest accuracy of 77.91%. Figure 4b is the plot of Receiver Operating Characteristics (ROC) *w.r.t.* different κ. The ROC space takes the true positive rate (TPR) as vertical axis and false positive rate (FPR) as horizontal axis, a better recognition performance is more closer to the upper left corner. So we can see that SemiTMC-GMM (red markers) obtains a better performance than the one of TMC-GMM (cyan markers). Table 1 shows the sensitivity, specificity, F1 score, and Matthews correlation coefficient (MCC) of each individual activity. Particularly for the sensitivity of each individual activity, it equals to the accuracy of corresponding activity. Activities D1A1 to D1A3 are recognized with relatively poor performance, it is because that these three activities are all walking and are very easily misclassified.

As reported in [20], the classifiers of kNN, SVM and decision tree are tested on SDA dataset using all the five sensors. The accuracies are 78.97%, 84.03%, and 84.63%, respectively. In [21], the authors used SDA dataset and showed single sensor recognition accuracy of four classifiers: kNN, decision tree, discriminant analysis, and Naive Bayes. Specifically for the right leg sensor that is used in our paper, the four classifiers obtained accuracies of 81.72%, 78.78%, 87.03%, and 76.93%. Therefore, we can state that SemiTMC-GMM outperforms the generic classifiers like kNN, SVM, decision tree, and Naive Bayes, and obtains a similar performance of discriminant analysis. On the other hand, the authors in [31] used CNN to recognize human daily activities in OPPORTUNITY dataset [32], which contains activities, such as open (close) door, open (close) drawer, clean table, and drink cup. They obtained an accuracy of 85.8% by using 23 body-worn sensors, 12 object sensors, and 21 ambient sensors. In addition, for the OPPORTUNITY dataset, [18] used CNN to obtain an accuracy of 77.99% by using the body-worn sensors only, while in [33], CNN obtained an accuracy of 93.75% on six activities: walking, stair ascent, stair descent, sitting, standing, and laying. Because of the prevalent CNNs can generate high dimensional features that suit for the recognition task, then CNNs may probably be suited for sophisticated activities. But it requires a huge quantity of data to train the network, and it is difficult to make CNN work for an adaptive on-line scenario. So, maybe CNN could obtain higher accuracy than SemiTMC-GMM, but we still believe that our proposed model is competent in some scenarios.

### 5.2. LMFIMU Dataset

**Figure 5:**
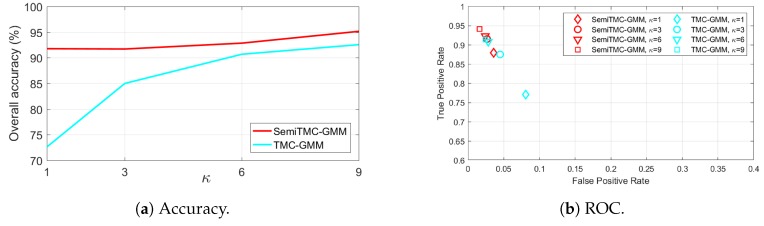
The batch mode recognition performance of the Locomotion of Foot-mounted IMU (LMFIMU) dataset, SemiTMC-GMM, and TMC-GMM models, according to different GMM mixture number *κ*.

For this dataset, the size of sliding window for computing features is set to 15, and it is also determined by our experience. Firstly, the batch mode recognition is performed using LOOCV strategy. Figure 5a shows the recognition accuracy *w.r.t.* different κ. The accuracy of SemiTMC-GMM when κ=9 is 95.16%, while the one of TMC-GMM is 92.57%. Meanwhile, the choice of κ has less impact on accuracy for SemiTMC-GMM. The recognition accuracy obtained by TMC-HIST is 80.42%, which is lower than the ones of TMC-GMM and SemiTMC-GMM when κ>1. The ROC shown in Figure 5b implies that SemiTMC-GMM outperforms the TMC-GMM in a general view. Table 2 shows the sensitivity, specificity, F1 score, and MCC of each individual activity. By comparing the batch mode recognition shown in Table 1 and Table 2, both TMC-GMM and SemiTMC-GMM outperform TMC-HIST. It means that considering the observation correlation improves the recognition performance.

**Figure 6:**
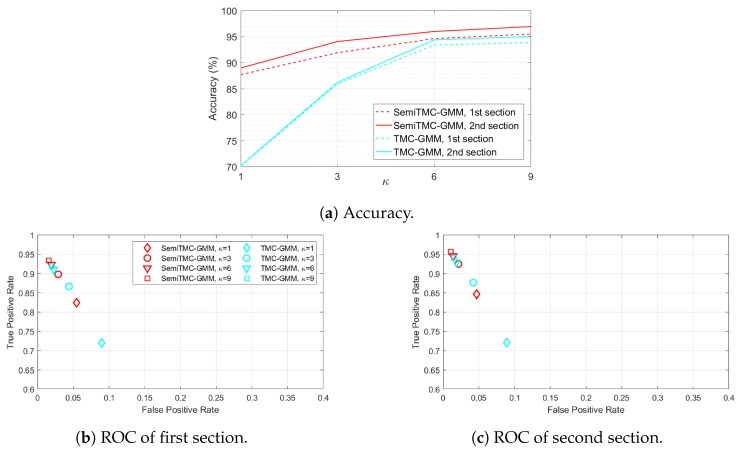
The on-line mode recognition performance of the two experiment sections in LMFIMU dataset, according to different GMM mixture number *κ*.

As a matter of fact, Figure 4a, Figure 5a and Figure 6a show that introducing semi-Markov structure into the TMC model can improve the accuracy. Meanwhile, using GMM with κ>1 also improves the recognition significantly. But it does not mean that using a larger κ allows higher accuracy to be achieved. In Figure 4a, the accuracy when κ=9 is slightly lower than that obtained when κ=6, it is because the observation of SDA dataset is more closer to a GMM mixture of 6 densities. A too large value of κ may probably lead to an overfitting problem. It is sure that κ can be automatically acquired through the methods, such as BIC [34] and AIC [35], to make κ consistent with different activities. For simplification in this paper, we manually set κ to 6 for all the activities based on the experimental results.

Then, the on-line EM algorithm is performed to validate the adaptive on-line recognition performances. The proposed algorithm is implemented in MATLAB code, running on a 64-bit system computer with 3.2GHz CPU and 32G RAM. In the dataset, the average experiment time is 32.33 min, while the computing time of SemiTMC-GMM when κ=1,3,6,9 are 9.72, 14.72, 21.53, and 27.65 min, respectively. Thus, using on-line EM is applicable in on-line scenarios. The window length $W_{l}$ for updating the parameters is set to 1000, which means that parameters are updated every 10 s.

Figure 6a shows the recognition accuracy obtained by LOOCV strategy. The solid lines are higher than the dashed lines which means that the on-line EM algorithm can improve the recognition performance. In addition, the GMM with κ>1 can significantly improve the accuracy. When κ=9, SemiTMC-GMM has an accuracy improved from 95.48% in the first section to 96.93% in the second section, TMC-GMM achieves an improvement from 93.83% to 95.04%. By contrast, in the adaptive on-line algorithm using TMC-HIST in our previous work, the accuracy was improved from 95.32% to 96.93%. However, this high accuracy is mainly because of the gait cycle complete detection in the adaptive on-line algorithm, which manually set the activity of all the samplings in one gait cycle to be identical. If the gait cycle complete detection is not used, TMC-HIST will fail in the on-line recognition, with the accuracies of 78.32% in the first section and 65.20% in the second section. Comparing SemiTMC (when κ=1) and TMC-HIST, we can conclude that semi-Markov structure is more robust for recognizing the hidden states which have sojourn time. Figure 6b,c show the ROC of the two experiment sections, SemiTMC-GMM obtains better performance than TMC-GMM in both the two sections, and SemiTMC-GMM obtains a bigger improvement from the first to second section than the other one. Therefore, the results indicate that both GMM density and semi-Markov structure improve the on-line recognition, and the combination the two improves the performance the most.

By comparing the two different sensor placements in SDA and LMFIMU dataset, the proposed algorithm shows that the sensor is not necessary to be placed at a specific place of the lower limb. In fact, the sensor can be placed in any position that implies the introduced gait phase and leg phase.

**Figure 7:**
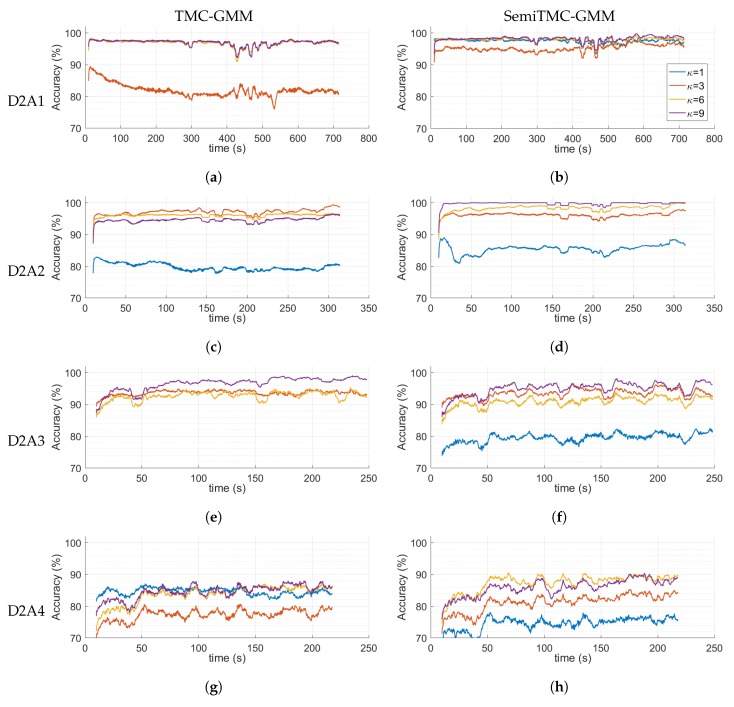
Recognition accuracy computed in the latest 10 s *w.r.t.* each activity of LMFIMU dataset. (**Left column**) TME-GMM; (**right column**) SemTMC-GMM.

In order to understand the dynamic performance of the parameter updating, Figure 7 shows the recognition accuracy computed during the latest 10 s. Notice that the accuracies when κ=1 are not displayed in Figure 7a,e because TMC obtains accuracies lower than 70% for D2A1 and D2A3. SemiTMC-GMM obtains a relatively fast convergence rate when κ equals to 6 and 9. The activities D2A1 and D2A2 reach high accuracy within 20 s in the first section of the experiment, 97.77% and 99.02%, respectively. By contrast, D2A3 and D2A4 are slower (take about 50 s) than the former two activities, and obtain lower accuracies of 92.04% and 89.48%, respectively. The main reason of this phenomenon is that the activity patterns of D2A3 and D2A4 vary much more differently among the subjects. But in a general view, we can still state that the on-line EM algorithm can dynamically improve the recognition accuracy to a reasonable level.

**Figure 8:**
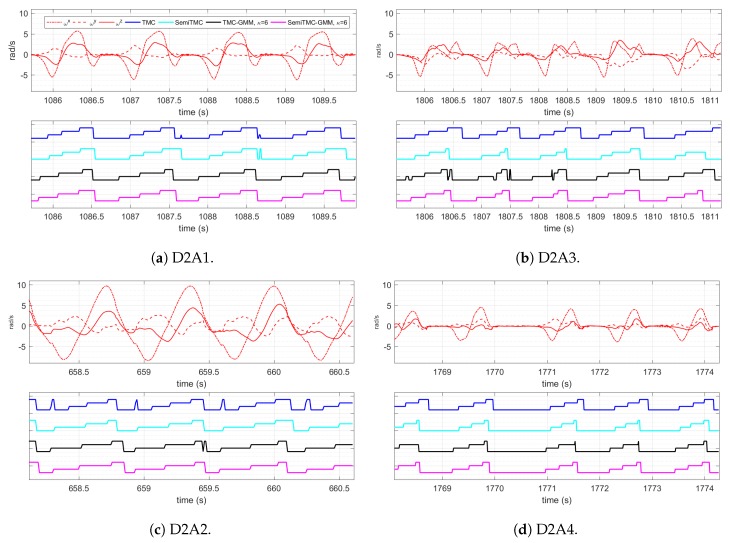
Estimated gait cycle of each activity. The blue, cyan, black, and magenta represent the gait obtained by TMC, SemiTMC, TMC-GMM, and SemiTMC-GMM, respectively.

Figure 8 displays the estimated gait cycles of each activity, when the model converged, obtained by TMC-GMM and SemiTMC-GMM, κ is set to 1 and 6. $\omega^{x}$, $\omega^{y}$ and $\omega^{z}$ are the sliding mean of angular rate along the three axes of sensor. The features are 12-dimensional, but here we only display the acceleration of along the three axes to show how the gaits proceed. Hence, the estimated gait cycles are displayed *w.r.t.* four models, i.e., TMC, SemiTMC, TMC-GMM, and SemiTMC-GMM. In fact, the gait phases or leg phases are introduced in the model to improve the recognition accuracy of the lower limb locomotion activity. The figure shows that SemiTMC-GMM obtains the most regular gait cycle, with no fluctuation in short period and no missing detection. As a consequence, the well estimated gait or leg cycle obtained from SemiTMC-GMM leads to a higher activity recognition performance.

## 6. Conclusions

In this paper, we proposed a wearable IMU-based algorithm for recognizing lower limb locomotion activities, with the help of introducing gait cycle or leg cycle into the model. The algorithm is based on the developed SemiTMC-GMM model, which better replicate the real motion. Our experiments show that semi-Markov structure and GMM density can better recover gait or leg cycles, which in return improve the activity recognition significantly. The adopted on-line EM algorithm can gradually improve the accuracy to a high level. The proposed algorithm is not only developed for the applications which require run-time activity recognition, but is also helpful to those applications that require gait cycles. For example, if using two sensors placed on both left and right legs of impaired people, then it is possible to develop a special SemiTMC-GMM model that detects their imbalanced gait phases. This can be beneficial for exoskeleton equipment to better assist impaired people in performing locomotion activities, by providing precise information to the equipment.

While there are still some limitations, the proposed algorithm only takes periodic lower limb locomotion into consideration; neither the static activity nor non-periodic lower limb locomotion activity is involved in our current work, such as standing and making turn. To distinguish static and motion activities, it is possible to include specific features into the observations. For example, standard deviation will be close to zero when a person is in static; otherwise, it will vary according to the motion. Distinguishing periodic and non-periodic can be accomplished by periodic pattern mining method, such as fast Fourier transform-based [36] and principle component analysis-based [37] approaches. Our future work will focus on adopting more types of activities, including static activity and non-periodic locomotion activities.

## Figures and Tables

**Table 1:** The sensitivity, specificity, F1 score, Matthews correlation coefficient (MCC) value of the batch mode recognition, for each activity of SDA dataset, using the sensor placed on right thigh. **Up**: TMC-HIST; **middle**: TMC-GMM when κ=6; and **down**: SemiTMC-GMM when κ=6.

		Activity					
**TMC-HIST**		**D1A1**	**D1A2**	**D1A3**	**D1A4**	**D1A5**	**D1A6**
	**Sensitivity**	0.4900	0.5463	0.6997	0.9017	0.7885	1.0000
	**Specificity**	0.9392	0.9883	0.9649	0.9839	0.9222	0.9939
	**F1 Score**	0.4687	0.6574	0.6837	0.8708	0.6057	0.9709
	**MCC**	0.4128	0.6461	0.6511	0.8587	0.5781	0.9684
		**D1A7**	**D1A8**	**D1A9**	**D1A10**	**D1A11**	**Total**
	**Sensitivity**	0.8308	0.7116	0.9489	0.9972	0.6618	0.7797
	**Specificity**	0.9911	0.9924	1.0000	1.0000	0.9813	0.9779
	**F1 Score**	0.8654	0.7966	0.9737	0.9986	0.7168	0.7826
	**MCC**	0.8535	0.7854	0.9715	0.9985	0.6936	0.7652
**TMC-GMM**		**D1A1**	**D1A2**	**D1A3**	**D1A4**	**D1A5**	**D1A6**
	**Sensitivity**	0.6784	0.6797	0.5483	0.9146	0.8980	1.0000
	**Specificity**	0.9322	0.9993	0.9866	0.9689	0.9465	0.9995
	**F1 Score**	0.5777	0.8059	0.6525	0.8164	0.7305	0.9978
	**MCC**	0.5353	0.8067	0.6382	0.8025	0.7151	0.9975
		**D1A7**	**D1A8**	**D1A9**	**D1A10**	**D1A11**	**Total**
	**Sensitivity**	0.8843	0.8917	0.8602	0.9876	0.8784	0.8383
	**Specificity**	0.9961	0.9940	0.9987	0.9998	0.9999	0.9838
	**F1 Score**	0.9197	0.9140	0.9184	0.9930	0.9348	0.8419
	**MCC**	0.9129	0.9059	0.9132	0.9923	0.9309	0.8319
**SemiTMC-GMM**		**D1A1**	**D1A2**	**D1A3**	**D1A4**	**D1A5**	**D1A6**
	**Sensitivity**	0.6672	0.7247	0.6182	0.9638	0.8767	0.9990
	**Specificity**	0.9457	0.9972	0.9860	0.9773	0.9563	0.9990
	**F1 Score**	0.6054	0.8273	0.7039	0.8752	0.7509	0.9944
	**MCC**	0.5644	0.8223	0.6862	0.8666	0.7327	0.9939
		**D1A7**	**D1A8**	**D1A9**	**D1A10**	**D1A11**	**Total**
	**Sensitivity**	0.9025	0.9410	0.8561	0.9956	0.9215	0.8606
	**Specificity**	0.9936	0.9922	0.9996	0.9994	1.0000	0.9860
	**F1 Score**	0.9175	0.9324	0.9208	0.9948	0.9590	0.8620
	**MCC**	0.9096	0.9255	0.9165	0.9943	0.9560	0.8516

**Table 2:** The sensitivity, specificity, F1 score, MCC value of the batch mode recognition, for each activity of LMFIMU dataset, using the sensor placed on right shoe. **Up**: TMC-HIST; **middle**: TMC-GMM when κ=9; and **down**: SemiTMC-GMM when κ=9.

		Activity				
		**D2A1**	**D2A2**	**D2A3**	**D2A4**	**Total**
**TMC-HIST**	**Sensitivity**	0.7007	0.9721	0.7705	0.9385	0.8454
	**Specificity**	0.9858	0.8931	0.9174	0.9595	0.9389
	**F1 Score**	0.8169	0.8258	0.6885	0.8596	0.7977
	**MCC**	0.7194	0.7833	0.6317	0.8382	0.7431
**TMC-GMM**	**Sensitivity**	0.9399	0.9475	0.9105	0.8590	0.9142
	**Specificity**	0.9720	0.9996	0.9512	0.9787	0.9754
	**F1 Score**	0.9547	0.9723	0.8327	0.8641	0.9060
	**MCC**	0.9130	0.9654	0.8044	0.8419	0.8812
**SemiTMC-GMM**	**Sensitivity**	0.9608	0.9829	0.9483	0.8749	0.9417
	**Specificity**	0.9831	0.9987	0.9634	0.9910	0.9841
	**F1 Score**	0.9713	0.9891	0.8799	0.9071	0.9368
	**MCC**	0.9445	0.9861	0.8600	0.8932	0.9210
